# Cardiac resynchronization therapy-defibrillator pocket infection caused by *Mycobacterium fortuitum*: a case report and review of the literature

**DOI:** 10.1186/s12872-019-1028-0

**Published:** 2019-03-05

**Authors:** Jun Zhu, Qingluan Yang, Junjie Pan, Haiming Shi, Bo Jin, Qiying Chen

**Affiliations:** 1 Department of Cardiology, Huashan Hospital of Fudan University, 12 Wulumuqi Zhong Road, Shanghai, 200040 China; 2 Department of infectious Diseases, Huashan Hospital of Fudan University, 12 Wulumuqi Zhong Road, Shanghai, 200040 China

**Keywords:** Cardiovascular implantable electronic devices infection, Rapid growing nontuberculous mycobacteria, *M. Fortuitum*, Case report

## Abstract

**Background:**

With the rising utilization of cardiovascular implantable electronic devices (CIEDs), infections secondary to device implantation are increasingly encountered. *Staphylococcus aureus* and coagulase-negative staphylococci are usually the predominant causative organisms. A CIED infection due to non-tuberculous mycobacteria (NTM) is extremely rare.

**Case presentation:**

A 68-year-old man was admitted to our hospital with a history of pain and swelling at his cardiac resynchronization therapy-defibrillator (CRT-D) pocket site, for 4 days. The CRT-D had been implanted 2 weeks prior. The exudate smear was positive for acid-fast bacilli and culture results revealed rapidly growing nontuberculous mycobacteria (RGM). After an urgent removal of the device followed by 1 year of antibiotic treatment, the patient was completely cured. A new device was finally implanted, 3 years later.

**Conclusions:**

Infections caused by nontuberculous mycobacteria following the implantation of cardiac devices are very rare. The typical manifestations of post-implantation CIED infections caused by RGMs include an early onset, with local redness, swelling, and spontaneous drainage. Systemic symptoms such as fever, chills, and fatigue are absent. *Mycobacterium fortuitum* is the most common species of RGM implicated in CIED infections, the manifestations of which usually appear within several weeks of the implantation procedure. An urgent removal of the device and appropriate antibiotic therapy are essential therapeutic measures. This is the first such reported case, in which the patient has been re-implanted with another device at the same site, after achieving a complete cure. We followed-up the patient for an additional 3 years and observed that the patient remained free of infection. Our case report shows that though an RGM infection is rare and difficult to treat, it can be completely cured. In addition, we demonstrated that it is subsequently possible to safely re-implant a CIED for the patient, at the same site.

## Background

In the last few decades, cardiac implantable electronic devices (CIEDs), which include permanent pacemakers (PMs), implantable cardioverter defibrillators (ICDs), and cardiac resynchronization therapy (CRT) devices have been widely utilized for the burgeoning aged population. As the survival benefits of CIED have been observed in clinical practice, their application is expected to increase. Despite the obvious benefits, the use of these implantable devices is also associated with serious complications. While improvements in implant technology have resulted in high success rates and lower occurrence of complications, an infection due to the device remains a major concern with the rate of secondary infection at about 1–2% [1]. An infection of the CIED significantly increases the morbidity, mortality, and healthcare costs of the procedure by prolonging the hospitalization time and mandating an aggressive management of the patient. An implant-associated infection can occur early or may be delayed for several months after the procedure. The main pathogens implicated in CIED infections are *Staphylococcus aureus* and coagulase-negative staphylococci [2]. A CIED infection caused by non-tuberculous mycobacteria (NTM) is extremely rare. To our knowledge, an infection has been reported in only 37 post-implantation cases, 28 of which were caused by a rapidly growing mycobacteria (RGM). Here, we present a case of CRT-D pocket infection caused by *Mycobacterium fortuitum*, which is the first reported case of its kind in China. It is also the first instance in which, after a complete recovery from the infection, a new device was re-implanted in the patient at the same site.

## Case presentation

A 68-year-old man had a history of chronic systolic heart failure and dilated cardiomyopathy, with a left ventricular ejection fraction of 32% and a left ventricular internal diastolic diameter of 81 mm. His electrocardiograph had revealed a sinus rhythm with a QRS duration of 140 ms along with a left bundle branch block morphology. He underwent a cardiac resynchronization therapy-defibrillator (CRT-D) device implantation. However, 2 weeks after the procedure, he was re-admitted to our hospital with a 4-day history of pain and swelling at the CRT-D pocket site, associated with scant serous drainage. Prior to this admission, he had undergone a skin incision and drainage at another hospital, but the procedure had failed to relieve his symptoms. The patient had a history of hypertension, a myocardial bridge in the left anterior descending coronary artery and diabetes mellitus (on irregular therapy). He also had a history of pulmonary tuberculosis 40 years previously and had completed the curative treatment course successfully, at the time. His regular medications included metoprolol, perindopril, torsemide, and amiodarone.

During re-admission, the patient did not have complaints of fever, chills, or fatigue. The physical examination was unremarkable, except for a 2-cm long, open incision on the upper left side of the chest, with mild localized edema over the CRT-D pocket. The skin around the incision was erythematous, and a small amount of scant serous discharge was noted on pressing the pocket site. Considering the likelihood of CRT-D infection, we collected the patient’s blood samples to perform cultures for aerobic and anaerobic bacteria. The samples of the exudate were also tested, using microscopic examination and culture, not only for the usual causative bacteria but also for the rarer acid-fast bacilli. On initial microscopic examination, the exudate smear did not reveal any organisms. An echocardiogram showed no evidence of vegetation or thrombosis. Other laboratory test results, including a routine blood count and erythrocyte sedimentation rate, were within normal limits. The patient also tested negative for the human immunodeficiency virus (HIV) and the hepatitis B virus. He was started empirically on intravenous vancomycin and cefepime.

On the 3rd day of hospitalization, we performed a pocket reconstruction. The CRT-D was re-implanted below the pectoralis major muscle in the left pectoral region and connected with the pacing electrodes placed in the earlier pocket site through a subcutaneous tunnel. However, the incision site did not heal, and the localized edema worsened, with copious exudation from the drainage site. After the reoperation, the exudate was collected and cultured. Within 3 days, a smear test of the exudate revealed an acid-fast bacillus, and culture results showed a rapidly growing nontuberculous mycobacterium (RGM). As recommended by our infectious diseases department, the isolate was sent to the microbiology laboratory for a polymerase chain reaction test. An amplification of the bacterial 16S ribosomal RNA gene, helped us identify the bacterial species as *Mycobacterium fortuitum (M. fortuitum)*. The patient was therefore diagnosed with CRT-D infection due to *M. fortuitum*.

Once the causative organism was definitively identified, the patient’s therapeutic antibiotic regimen was empirically changed to intravenous clarithromycin, moxifloxacin, and amikacin. At the same time, an incision and debridement of the first pocket site was performed, and the entire device along with the leads were extracted, percutaneously. With dedicated wound care, the swab culture tests eventually yielded negative results and the incision healed well. After 2 months of administration of combined intravenous treatment, the patient was discharged. He was further administered oral clarithromycin and moxifloxacin for 1 year. He was continued to follow up for next 2 years. During 3 years, we checked the pocket site every 3 months and evaluated his cardiac function by echocardiography, as well as recorded the QRS complex width by ECG. He remained asymptomatic of incision infection but we found that the patient’s heart failure condition had gradually deteriorated (left ventricular ejection fraction decreased from 32 to 26%) and life-threatening rapid ventricular arrhythmias occurred even under the optimal drug treatment. The patient was then re-implanted with another CRT-D device at nearly the same previous site. The symptoms of heart failure were significantly improved. ECG showed the QRS complex width was decreased from 140 ms to 112 ms. Echocardiography showed the LVEF were increased to 45% after 6 months of the re-implantation. The patient has now remained free of infection over 4 years (Table 1).

**Table 1:** Timeline

2011.5.30	Implanted the cardiac resynchronization therapy-defibrillator device
2011.6.11	Onset of pain and swelling at his CRT-D pocket site
2011.6.15	Empirically anti-infective treatment by intravenous Vancomycin and Cefepime.
2011.6.18	Pocket reconstruction and CRT-D re-implanted below pectoralis major muscle at the left pectoral region.
2011.6.21	Incision was not healed and exudation was collected for culture
2011.6.23	Exudation smear was positive for acid-fast bacillus and culture results showed rapid growing nontuberculous mycobacteria(RGM)
2011.6.24	PCR amplification of the bacterial 16S ribosomal RNA gene, it was identified as *Mycobacterium fortuitum*.
2011.6.25	Entire CRT-D device and leads were removed
2011.6.25	Antibacterial treatment: Clarithromycin and Moxifloxacin and Amikacin for 2 months.
2011.8.25	Therapy with oral Clarithromycin and Moxifloxacin for one year.
2012.8	Stop antibacterial treatment
2013.8	Follow- up after one year
2014.8	Follow-up two years and received a new CRT-D device implantation.
2018.8	Follow-up four-years after new device implanted. No symptoms of any infection.

## Discussion and conclusions

With growing evidence that a CIED implant is of benefit to patients with chronic cardiac disease, the utilization of these devices has increased over the last few years. The incidence of CIED infection is therefore correspondingly on the rise. The reported rates of infection secondary to CIED implantation, differ across various studies, ranging from 0.13 to 19.9%. The most recent studies report a rate of infection between 1 and 2% [3, 4].

The risk factors for CIED infections include prior immunodeficient states, i.e. patients suffering from HIV infection, diabetes mellitus, and chronic renal insufficiency are at a greater risk. In addition, many studies have found a higher rate of infection associated with ICDs or CRT devices, nearly 2–5 times higher than that associated with PM [5, 6]. This may be due to the larger sizes of the ICD and CRT devices and the longer operative duration required for implanting them. The use of anticoagulants has been associated with a higher risk of pocket hematoma, which also exacerbates the risk of a generator pocket infection.

Gram-positive bacteria are the predominant causative pathogens of CIED infections. Coagulase-negative staphylococci and *Staphylococcus aureus* account for 70–80% of all infections following CIED implantations [7]. However, RGM infections in implanted cardiac devices are still very rare. We searched the PubMed database for any older studies (no specified start time), case reports, or reviews related to CIED infections, using several search strings that combined the names of the various NTM with the different implantable cardiac devices. To be more specific, we used the following free text terms: (rapid growing nontuberculous mycobacteria OR nontuberculous mycobacteria OR *Mycobacterium fortuitum* OR *Mycobacterium chelonae* OR *Mycobacterium abscessus* OR *Mycobacterium smegmatis* group OR *Mycobacterium mucogenicum*) AND (cardiovascular implantable electronic devices OR CIED OR pacemaker OR PM OR cardiac resynchronization therapy OR CRT OR cardiac resynchronization therapy-defibrillator OR CRT-D OR implantable cardioverter defibrillator OR ICD). A review published by Phadke et al. summarized 23 cases with CIED infection caused by RGM up to March 31, 2015 [8]. Including the cases in their study, we found records for a total of 27 older cases that were identified as CIED infections due to RGM [8–30]. This makes our patient only the 28th recorded case of CIED infection, caused by an RGM species.

RGM comprise approximately half of the currently known mycobacterial species and are divided into five major groups: *Mycobacterium fortuitum*, *M. chelonae/M. abscessus* complex*, M. smegmatis, M. mucogenicum,* and the pigmented RGM [31]. The RGM species are universal organisms that exist in soil, food, natural and tap water, as well as in various plants and animals [32, 33]. They can grow in municipal water systems as well as distilled water and are resistant to sterilizers, antiseptics, and other standard disinfectants [33, 34]. The most commonly encountered RGM species in clinical practice are *M. fortuitum* and *M. abscessus/M. chelonae*. Of the recorded 28 CIED infections caused by RGM (Table 2), 17 (61%) were reportedly caused by *M. fortuitum.* We found 4 reported cases each, of CIED infections caused by the *Mycobacterium abscessus/chelonae* complex and the *Mycobacterium smegmatis* group. Only 2 patients were found to be infected by the *Mycobacterium mageritense* group and the pigmented RGM were identified only in 1 case. While 16 (60%) patients had infections associated with permanent PMs and 9 patients presented with ICD infection, we found no previous reports of CIED following a CRT-D implantation procedure, which makes our case report the first of its kind with respect to the type of device involved. Of the 28 patients, 21 patients (75%) developed an infection in < 6 months after the device implantation, of which, 12 patients were affected within 4 weeks. Therefore, we can surmise that the patients presenting with an early-onset infection are likely to have contracted it primarily during the implantation procedure and not as a secondary infection through the bloodstream. Only 4 of the 28 cases developed clinical manifestations of an infection > 1 year after device implantation. To our knowledge, *M. fortuitum* is not considered a normal skin commensal. These infections are considered to have been postoperatively contracted from an environmental source. We observed that 13 (46%) of the 28 reported patients with an RGM infection had other associated infections caused by mycobacteria. While 3 of these 13 patients were found to fulfil the clinical criteria for infective endocarditis through echocardiographic findings, in 1 patient, the causative mycobacterium was isolated on culture postoperatively, thus fulfilling the pathologic criteria of diagnosis.

**Table 2:** Clinical and demographic information for published cases of cardiac device infections due to rapidly growing mycobacteria

Reference	Age (years) /sex	Onset^a^	Type	Bacteremia^b^	IE^c^	Organism	Device Removal	Antibiotics Treatment/Duration	Outcome
*Mycobacterium fortuitum* group									
Verghese, 1998	74/M	13 d	PPM	NR	NR	*Mycobacterium fortuitum* / *Mycobacterium chelonae*	Yes	Fluoroquinolone + Aminoglycoside /1 mo	Cured
Sharma, 2005	62/F	6mo	PPM	Yes	Yes	*Mycobacterium fortuitum*	Yes	Clarithromycin + Ciprofloxacin/4wk, Doxycycline+ Ciprofloxacin /24wk	Cured
Short,2005	74/M	6 wk	ICD	Yes	NR	*Mycobacterium fortuitum*	Yes	Clarithromycin + Ciprofloxacin /6wk	Cured
Hemmersbach-Miller,2005	72/M	2wk	PPM	No	No	*Mycobacterium fortuitum*	Yes	Ciprofloxacin + Aminoglycoside /2wk, Ciprofloxacin /6mo	Cured
Hemmersbach-Miller,2005	61/M	17mo	ICD	No	No	*Mycobacterium fortuitum*	Yes	Levofloxacin /≥1 yr	Cured
Pastor, 2006	80/M	18 d	PPM	Yes	No	*Mycobacterium fortuitum*	No	Ciprofloxacin + Clarithromycin/6 wk	Cured
Giannella, 2007	84/F	2 mo	PPM	No	No	*Mycobacterium fortuitum*	Yes	Levofloxacin/3 mo	Cured
Siu, 2007	78/F	6 mo	PPM	Yes	No	*Mycobacterium fortuitum*	Yes	Levofloxacin + Clarithromycin/6 mo	Cured
Tam, 2007	78/F	4 mo	PPM	Yes	NR	*Mycobacterium fortuitum*	Yes	Levofloxacin + Clarithromycin/6 mo	Cured
Al Soub H, 2009	15/F	7 wk	PPM	Yes	No	*Mycobacterium fortuitum*	Yes	Ciprofloxacin + Clarithromycin/6 mo	Cured
Van Duin,2010	78/M	NR	PPM	Yes	NR	*Mycobacterium fortuitum*	Yes	Ciprofloxacin + Clarithromycin/26wk	Cured
Sharma,2012	43/M	4 yr	ICD	Yes	Yes	*Mycobacterium fortuitum*	Yes	Ciprofloxacin + Clarithromycin+ Aminoglycoside	Died
Amraoui,2012	75/M	1 yr	PPM	Yes	No	*Mycobacterium peregrinum*	Yes	Ciprofloxacin + Clarithromycin/mo	Cured
Yuhning,2012	56/M	4mo	ICD	Yes	No	*Mycobacterium fortuitum*	No	Cephalexin, Clarithromycin+ Moxifloxacin/ 2 wk	Died
Varun,2016	60/M	6wk	ICD	Yes	No	*Mycobacterium fortuitum*	Yes	Ciprofloxacin + Clarithromycin+ Cefoxitin	Died
Menfil Orellana-Barriors,2017	59/F	3wk	ICD	No	No	*Mycobacterium fortuitum*	Yes	Levofloxacin + Clarithromycin/23 wk	Cured
2018^d^	68/ F	10 d	CRT	No	No	*Mycobacterium fortuitum*	Yes	Clarithromycin + Moxifloxacin/12 mo	Cured
*Mycobacterium chelonae/abscessus* complex									
Cutay, 1998	68/M	19 yr	PPM	NR	NR	*Mycobacterium abscessus*	Yes	Clarithromycin + Cefoxitin + Amikacin/5 wk	Died
Kessler, 2004	53/F	2 wk	ICD	NR	NR	*Mycobacterium abscessus*	Yes	Clarithromycin/6 mo	Cured
Simmon KE,2007	43/F	11mo	PPM	NR	NR	*Mycobacterium massiliense*	Yes	Clarithromycin/6 mo	Cured
Hooda,2014	63/M	NR	PPM	No	Yes	*Mycobacterium chelonae*	Yes	Clarithromycin + Levofloxacin + Aminoglycoside />2mo	Cured
*Mycobacterium smegmatis* group									
Toda H,2006	86/M	16d	PPM	Yes	NR	*Mycobacterium goodii*	Yes	Minocycline + Aminoglycoside /2wk	Cured
Chrissoheris MP,2008	85/M	<7d	ICD	No	No	*Mycobacterium goodii*	Yes	Trimethoprim/Sulfamethoxazole /8wk	Cured
Marchandin H, 2009	23/M	8d	PPM	No	No	*Mycobacterium goodii*	No	Doxycycline +Fluoroquinolone /6mo	Cured
David,2016	74/F	1mo	PPM	No	No	*Mycobacterium goodii*	Yes	Ciprofloxacin + Doxycycline/6mo	Cured
*Mycobacterium mageritense* group									
Tam, 2007	77/F	3 wk	PPM	NR	NR	*Mycobacterium mageritense*	No	Levofloxacin/6 mo	Cured
Masato Fukunaga, 2016	59/F	14d	ICD	Yes	No	*Mycobacterium mageritense*	Yes	Ciprofloxacin + Clarithromycin/ 1 yr	Cured
The pigmented rapid growing species									
Karnam S, 2011	73/F	1 mo	ICD	No	NR	*Mycobacterium phlei*	Yes	Trimethoprim/Sulfamethoxazole +Doxycycline /12 mo	Cured

*ICD* implantable cardioverter-defibrillator, *PPM* permanent pacemaker, *NR* not reported, *IE* infective endocarditis. *M* Male, *F* Female, *yr.* year, *mo* month, *wk*. week, *d* day

^a^Time since most recent device manipulation

^b^Defined as positive blood culture, acid fast stain

^c^Transthoracic or transesophageal echocardiographic findings

^d^The patient described in this article

The CIED infections are generally categorized as superficial or deep infections. The superficial infections like a generator pocket infection are commoner than the deep infections affecting the cardiac leads or valves. The most common manifestations of a generator pocket infection are fever, pain at the generator site, and wound discharge. Therefore, when an operated patient is observed to have slow wound healing or has a history of the implant site being re-opened and re-manipulated for any reason, an RGM infection should be strongly suspected (especially in an immunocompromised host). However, while a patient with an RGM may present with localized redness, swelling, and with spontaneous drainage, the typical systemic symptoms of fever, chills and fatigue are absent. The wound exudate is usually thin and watery but may sometimes be thick and purulent [33]. An echocardiography along with cultures of the patient’s blood, device pocket tissue, and leads are essential for the diagnostic evaluation. The tissue cultures of samples obtained during the surgery are more sensitive than swab cultures [35].

While the management of RGM infections must be tailored individually, an urgent device removal followed by antimicrobial therapy forms the mainstay of treatment in all cases. An American Heart Association statement recommended a complete removal of all hardware (Class I evidence) from patients, even in the absence of signs of systemic infection. An incomplete removal of CIED has resulted in higher infection relapse rates. These organisms can form biofilms, which make them difficult to eradicate with antimicrobial drug therapy alone. In our case, the device was urgently extracted from the patient and 3 years later, once he was disease-free, he was re-implanted with another device, which suggests the possibility of re-implantation after cure in other such patients.

**Fig. 1:**
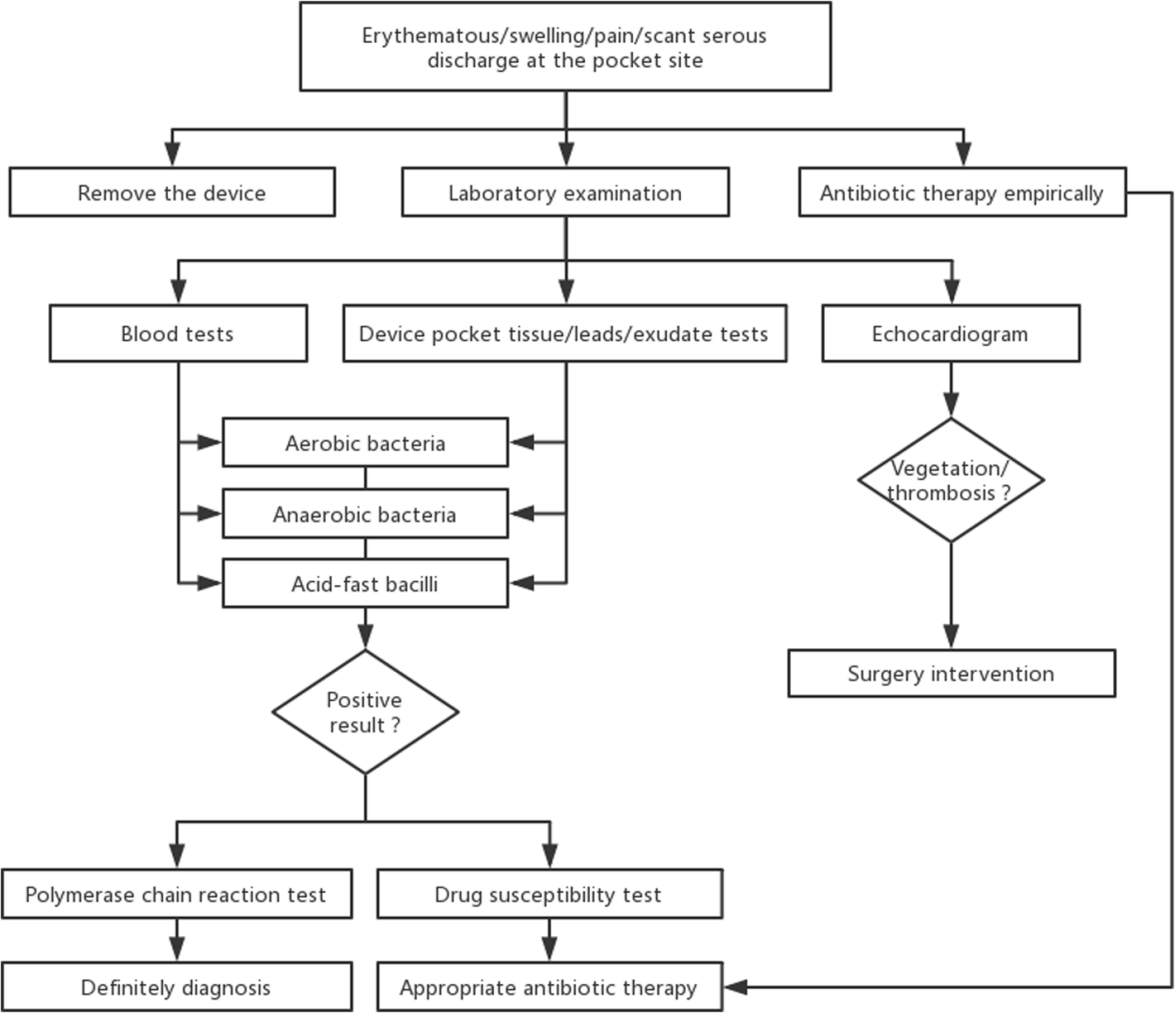
A recommended diagnostic and therapeutic of CIED infections flow chart

After removal of the device, antibiotic treatment is essential. An antimicrobial drug susceptibility test is important for the selection of the appropriate antibiotic therapy. A recommended diagnostic and therapeutic flow chart is shown in Fig. 1. *M. fortuitum* is usually found to be susceptible to amikacin, cefoxitin, sulfonamides, ciprofloxacin, imipenem and moxifloxacin, with about half of the isolates susceptible to doxycycline [36]. Approximately 80% of *M. fortuitum* isolates can be inhibited by clarithromycin. Among newer antimicrobials, linezolid has been shown to have in vitro activity against the *M. fortuitum* group [37]. Unfortunately, most of the effective antimicrobial drugs for RGM have potentially adverse proarrhythmic effects. In our patient, we regretted the fallacy of prescribing antimicrobials empirically, without an initial drug susceptibility test. The treatment duration is dependent on the completeness of device removal, the greater the number of components of a device left in situ, the longer the duration of treatment. A longer duration of antibacterial therapy is necessary due to the propensity of the organisms towards biofilm formation. In 16 of the 28 recorded cases, the antibiotic treatment duration was ≥6 months. The optimal timing of device replacement after removal is not defined, as it may vary individually, depending on the extent of infection in each patient. It is recommended that the device should be replaced on the contralateral side from the older infected site [35]. In our patient, we implanted a new CRT-D, 3 years after the removal of the older infected device. As a generator placed on the right side could decrease the effect of defibrillation, the new device was implanted on the same side (left side) as before. Over 3 years of follow-up, the patient has shown no recurrence of an RGM infection.

Preventive measures are extremely important to minimize the risk of infection. A microbial contamination can occur during implantation, post-implantation or can occur secondary to an erosion of the device through skin. Several observational studies have demonstrated that almost half of the CIED infections can be prevented by prophylactic antibiotic therapy administered in the perioperative period [38]. The risk of infection is proportional to the duration of the implantation procedure. Therefore, we can reduce risk of CIED infections by strict aseptic manipulation, perioperative antibiotic prophylaxis, and by limiting the operative duration. Furthermore, temporary pacing should be avoided, if possible, because temporary transvenous pacing is associated with an increased risk of infection [39]. The utilization of newer devices such as subcutaneous defibrillators, leadless pacemakers and bio-absorbable antibacterial envelopes may also contribute to decreasing the incidence of infection in patients with CIED [40–43].

The infections occurring after implantation of cardiac devices and caused by nontuberculous mycobacteria are rare. However, with an increased utilization of cardiac implant devices in patients, the occurrence of consequent infections is also expected to increase. Our patient presented with typical manifestations of a post-implantation CIED infection caused by RGMs, including early onset with localized implant site redness, swelling, spontaneous drainage, and without any systemic symptoms. After identifying *M. fortuitum*, as the causative organism, the device was completely removed at once, and the appropriate antibiotic therapy was administered. The treatment had a successful outcome as our patient was completely cured. The limitation of our case was that we prescribed antimicrobials empirically, without an initial drug susceptibility test. Furthermore, we implanted another device in the same patient after 3 years, which is an encouraging indication that re-implantation after the resolution of infection in other such patients may be possible. However, further investigation is required to determine the suitable time interval between the successful completion of curative treatment and re-implantation.

## Abbreviations

CIED: Cardiovascular implantable electronic devices
CRT-D: Cardiac resynchronization therapy-defibrillator
ICD: Implantable cardioverter defibrillator
NTM: Non-tuberculous mycobacteria
PM: Permanent pacemaker
RGM: Rapid growing nontuberculous mycobacteria
